# Low-Complexity High-Order Propagator Method for Near-Field Source Localization

**DOI:** 10.3390/s19010054

**Published:** 2018-12-23

**Authors:** Jianzhong Li, Yide Wang, Cédric Le Bastard, Zongze Wu, Shaoyang Men

**Affiliations:** 1 School of Automation, Guangdong University of Technology, Guangzhou 510006, China; jianzhong.li@gdut.edu.cn (J.L.); zzwu@gdut.edu.cn (Z.W.); 2 Institut d’Electronique et Télécommunications de Rennes (IETR), Université de Nantes, UMR CNRS 6164, Rue Christian Pauc BP 50609, 44306 Nantes, France; yide.wang@univ-nantes.fr (Y.W.); Cedric.Lebastard@cerema.fr (C.L.B.); 3 Cerema, Project-Team ENDSUM, 49136 Les Ponts de Cé, France; 4 School of Medical Information Engineering, Guangzhou University of Chinese Medicine, Guangzhou 510006, China

**Keywords:** propagator, near-field, high-order, cumulant

## Abstract

In this paper, an efficient high-order propagator method is proposed to localize near-field sources. We construct a specific non-Hermitian matrix based on the high-order cumulant of the received signals. With its columns and rows, we can obtain two subspaces orthogonal to all the columns of two steering matrices, respectively, with which the estimation of the directions of arrival (DOA) and ranges of near-field sources can be achieved. Different from other methods, the proposed method needs only one matrix for estimating two parameters separately, therefore leading to a smaller computational burden. Simulation results show that the proposed method achieves the same performance as the other high order statistics-based methods with a lower complexity.

## 1. Introduction

Source localization plays an important role in the array signal processing area. According to the distance between the receiver array and the sources, source localization can be classified as far-field and near-field. In the far-field case, the impinging signal wavefront is considered as a plane, and its direction of arrival (DOA) is enough to localize the source [1]. However, this assumption fails to hold in the near-field scenario. More specifically, the wavefront of an impinging signal is spherical [2,3]. Both the range and DOA are required to localize the source. It is worth noticing that near-field source localization can be applied in a wide range of scenarios, including, but not limited to, radar, sonar, and electronic surveillance [4,5].

There are already many methods to achieve the goal of source localization, like beam-forming [6,7], MUSIC [8], root-MUSIC [9], ESPRIT [10,11,12], sparse-reconstruction-based methods [13], and others. However, these methods are proposed only for the far-field case. The most direct way to localize a near-field source is to extend the basic MUSIC algorithm to estimate the range and DOA simultaneously, which is referred to as the two-dimensional (2D) MUSIC method [14]. Similarly, the basic ESPRIT method can be extended to the 2D ESPRIT algorithm [15]. It is worth observing that a large computational complexity is required for the MUSIC-based methods due to the 2D search, which is implemented in both the range and direction domains. In order to reduce the complexity, a second-order statistics MUSIC method is proposed in [16] to transform the 2D search into several one-dimensional (1D) ones. However, this reduction of complexity is at the cost of aperture loss. In [17], the DOAs are firstly estimated by using a focusing technique, but it requires a beamforming-based pre-estimation technique. In [18], the near-field sources are located with three second-order matrices, estimating three parameters through rooting methods. However, besides suffering from aperture loss, an extra pairing algorithm is also required. The method proposed in [19] is also based on the second-order statistics, but without eigenvalue decomposition (EVD), thus achieving a better computational efficiency.

In recent years, the application of a high-order cumulant has been drawing many scholars’ attention [20,21,22]. A high-order cumulant can achieve higher degrees of freedom than the second-order statistics, allowing more possible construction forms of the desired matrices [23]. Besides, the excellent resistance against Gaussian noise of a high-order cumulant is also an important advantage [24]. The ESPRIT-like method proposed in [23] constructs several different matrices to estimate two position parameters without any exhaustive search, therefore achieving a high efficiency. However, this kind of method needs an extra pairing algorithm to match the estimated parameters. The method proposed in [25] improves these ESPRIT-like methods by avoiding the parameter matching procedure. Based on these high-order statistics methods, a further improvement (modified ESPRIT-like) was proposed in [26], which requires only one matrix without pairing steps.

In [27], a modified 2D MUSIC method was proposed. This method utilizes the characteristics of high-order statistics and does not suffer from aperture loss. In [28], a mixed-order statistics MUSIC (MOS) was proposed to improve the method of [27]. However, for almost all the existing methods, Hermitian matrices are adopted for the parameter estimation, which indeed are not necessary. For the modified MUSIC methods, the construction of two Hermitian matrices is necessary, and the time-consuming EVD must be implemented twice. These disadvantages result in a very high computational complexity and make the implementation of the methods very complicated.

Propagator-based methods require neither singular-value decomposition (SVD) nor EVD [29,30]. Considering the high degrees of freedom and resistance to colored Gaussian noise of a high-order cumulant, we propose a high-order propagator method that requires only one matrix and no EVD, therefore significantly reducing the computational complexity. Firstly, we construct a specific non-Hermitian cumulant matrix. Then, by exploiting the properties of its columns, a subspace orthogonal to the columns of one steering matrix is built. The DOAs can be directly estimated based on this orthogonality. For the range estimation, another subspace orthogonal to the columns of another steering matrix is built with the rows of the cumulant matrix. The ranges can then be estimated one by one for each estimated DOA.

The rest of this paper is organized as follows. Section 2 presents the signal model and some common assumptions. In Section 3, the proposed method is described in detail. The complexity analysis is also given to illustrate the improvement of the proposed method. In Section 4, several simulations are provided. At last, the conclusion of the whole paper is made in Section 5.

In this paper, *T* represents the transpose operation, *H* the conjugate transpose, and * the complex conjugate. A bold capital letter symbolizes a matrix, and a bold letter in lowercase stands for a vector, such as A and a, respectively.

## 2. Signal Model

**Figure 1:**
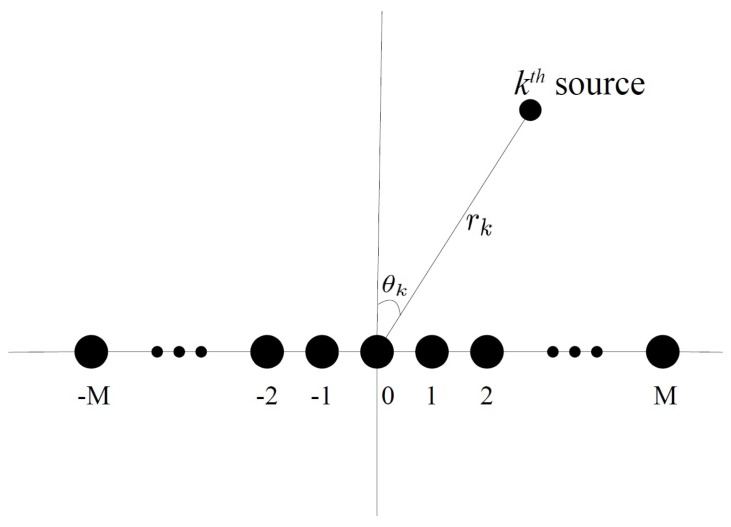
Near-field source localization with ULA.

In this paper, we consider a uniform linear array (ULA) model consisting of 2M+1 sensors as shown in Figure 1. The inter-element spacing is *d*. Assume that *K* near-field narrow-band signals impinge on the ULA. The sampling frequency is also assumed to be normalized. The output of the mth(m∈[−M,M]) sensor can be expressed as:

(1) $$y_{m}\left(t\right)=\sum_{k=1}^{K}s_{k}\left(t\right)e^{j\varphi_{mk}}+n_{m}\left(t\right),t=1,2,\ldots ,T,$$

where $s_{k}\left(t\right)$ is the kth source signal received at the zeroth sensor, *T* represents the number of snapshots, $n_{m}\left(t\right)$ is the additive Gaussian noise at the mth sensor, which may be colored, and $\varphi_{mk}$ is the phase difference expressed as [17]:

(2) $$\begin{array}{ccc} \varphi_{mk} & = & \frac{2\pi }{\lambda }\left(\sqrt{r_{k}^{2}+{\left(md\right)}^{2}-2r_{k}md\sin\theta_{k}}-r_{k}\right)\\ & \approx & \omega_{k}m+\phi_{k}m^{2}, \end{array}$$

where:

(3) $$\begin{array}{c} \omega_{k}=-\frac{2\pi d}{\lambda }\sin\theta_{k}, \end{array}$$

(4) $$\begin{array}{c} \phi_{k}=\frac{\pi d^{2}}{\lambda r_{k}}\cos^{2}\theta_{k}, \end{array}$$

with λ being the wavelength of the source signal, satisfying λ≥4d, $r_{k}$ the range of the kth source, and $\theta_{k}$ the corresponding DOA.

The received signal can also be expressed in the following matrix form:

(5) y(t)=A(θ,r)s(t)+n(t),

where y(t) is the (2M+1)×1 received signal vector:

(6) $$y\left(t\right)={\left[y_{-M}\left(t\right),y_{-M+1}\left(t\right),\ldots ,y_{M}\left(t\right)\right]}^{T},$$

s(t) is the K×1 signal vector from the *K* sources:

(7) $$s\left(t\right)={\left[s_{1}\left(t\right),s_{2}\left(t\right),\ldots ,s_{K}\left(t\right)\right]}^{T},$$

A(θ,r) is the steering matrix:

(8) $$A\left(\theta ,r\right)=[a\left(\theta_{1},r_{1}\right),a\left(\theta_{2},r_{2}\right),\ldots ,a\left(\theta_{K},r_{K}\right)],$$

$a(\theta_{k},r_{k})$ is the (2M+1)×1 steering vector:

(9) $$\begin{array}{c} a\left(\theta_{k},r_{k}\right)={\left[e^{j[\left(-M\right)\omega_{k}+{\left(-M\right)}^{2}\phi_{k}]},\ldots ,e^{j(M\omega_{k}+M^{2}\phi_{k})}\right]}^{T}, \end{array}$$

and n(t) is the (2M+1)×1 noise vector:

(10) $$n\left(t\right)={\left[n_{-M}\left(t\right),n_{-M+1}\left(t\right),\ldots ,n_{M}\left(t\right)\right]}^{T}.$$

Without loss of generality, we make the following assumptions, which are the same as those in [16,17,18,19,20,21,22,23,24,25,26,27,28]:

(1) The kurtosis of the source signal is non-zero.

(2) All the DOAs are different from each other.

(3) The Gaussian noise $n_{m}\left(t\right)$ is independent of the source signals, and the *K* source signals are independent of each other.

(4) The number of sources *K* has already been estimated [19], which can be achieved through various methods, such as the Akaike information criterion (AIC) [31], the Kullback–Leibler information criterion (KIC) [32], and Rissanen’s minimum description length (MDL) principle [33]. Furthermore, *K* is smaller than the number of sensors.

## 3. Proposed Method

In [34], we proposed a simplified high-order MUSIC method (SHO), where only one non-Hermitian matrix is constructed and one EVD is applied to localize near-field sources. The computational complexity of SHO has been proven to be lower than that of MOS, but the constructed non-Hermitian matrix should be diagonalizable. In this paper, we propose a high-order propagator-based method (HOP) that can achieve the sequential estimation of the DOA and range of near-field sources with only one matrix and without the application of EVD. The complexity therefore is greatly reduced. Furthermore, the proposed HOP method can still work correctly even if the non-Hermitian matrix is not diagonalizable.

### 3.1. DOA Estimation

The fourth-order cumulant of the signal is applied in this paper for the construction of a (2M+1)×(2M+1) matrix. In particular, the cumulant of Gaussian noise is always zero when the statistic order is greater than 2 [24]:

(11) $$cum\{n_{m}\left(t\right),n_{n}^{*}\left(t\right),n_{p}\left(t\right),\ldots \}=0.$$

Therefore, the fourth-order cumulant shows an excellent resistance to the Gaussian noise, no matter whether the noise is colored or white. For the sake of simplicity, the noise in cumulant equations will be ignored in the sequel, and we will concentrate on the source signal.

With Assumptions (1) and (3) in Section 2, the fourth-order cumulant of the received signal can be written as [16,23,27,28]:

(12) $$\begin{array}{cc} & cum\{y_{m}\left(t\right),y_{n}^{*}\left(t\right),y_{p}\left(t\right),y_{q}^{*}\left(t\right)\}\\ & =E[y_{m}\left(t\right)y_{n}^{*}\left(t\right)y_{p}\left(t\right)y_{q}^{*}\left(t\right)]\\ & -E\left[y_{m}\left(t\right)y_{n}^{*}\left(t\right)\right]E\left[y_{p}\left(t\right)y_{q}^{*}\left(t\right)\right]\\ & -E\left[y_{m}\left(t\right)y_{p}\left(t\right)\right]E\left[y_{n}^{*}\left(t\right)y_{q}^{*}\left(t\right)\right]\\ & -E\left[y_{m}\left(t\right)y_{q}^{*}\left(t\right)\right]E\left[y_{n}^{*}\left(t\right)y_{p}\left(t\right)\right]\\ & =\sum_{k=1}^{K}c_{4s_{k}}e^{j[\left(m-n+p-q\right)\omega_{k}+\left(m^{2}-n^{2}+p^{2}-q^{2}\right)\phi_{k}]}, \end{array}$$

$$\begin{array}{cc} & cum\{s_{m}\left(t\right),s_{n}^{*}\left(t\right),s_{p}\left(t\right),s_{q}^{*}\left(t\right)\}\\ & =\left\{\begin{array}{cc} c_{4s_{k}}, & \left(m=n=p=q=k\right)\\ 0, & \left(others\right) \end{array}\right. \end{array}$$

where $c_{4s_{k}}$ is the fourth-order cumulant of $s_{k}$.

A cumulant matrix is constructed with the following entries:

(13) $$\begin{array}{ccc} C(\bar{m},\bar{n}) & = & cum\{y_{m}\left(t\right),y_{-m}^{*}\left(t\right),y_{0}\left(t\right),y_{n}^{*}\left(t\right)\}\\ & = & \sum_{k=1}^{K}c_{4s_{k}}e^{j2\omega_{k}m}e^{-j(\omega_{k}n+\phi_{k}n^{2})}, \end{array}$$

where:

(14) $$\bar{m}=M+m+1,$$

(15) $$\bar{n}=M+n+1,$$

(16) m,n∈[−M,M].

The cumulant matrix **C** can be written in the matrix form as:

(17) $$C=A_{1}\left(\theta \right)C_{4s}A_{2}^{H}\left(\theta ,r\right),$$

where $C_{4s}$ is a diagonal matrix with the diagonal entries being $c_{4s_{1}},c_{4s_{2}},\ldots ,c_{4s_{K}}$:

(18) $$C_{4s}=\left[\begin{array}{ccccc} c_{4s_{1}} & 0 & 0 & \ldots & 0\\ 0 & c_{4s_{2}} & 0 & \ldots & 0\\ \vdots \\ 0 & 0 & 0 & \ldots & c_{4s_{K}} \end{array}\right].$$

Assume that $a_{1}\left(\theta_{k}\right)$ and $a_{2}\left(\theta_{k},r_{k}\right)$ are the $k^{\mathrm{th}}$ columns of the steering matrices $A_{1}\left(\theta \right)$ and $A_{2}\left(\theta ,r\right)$, respectively, which are (2M+1)×1 vectors given by:

(19) $$a_{1}\left(\theta_{k}\right)={\left[e^{j2\left(-M\right)\omega_{k}},\ldots ,e^{j2M\omega_{k}}\right]}^{T}$$

and:

(20) $$\begin{array}{cc} a_{2}\left(\theta_{k},r_{k}\right)=[ & e^{j[\left(-M\right)\omega_{k}+{\left(-M\right)}^{2}\phi_{k}]},\ldots ,\\ & e^{j(M\omega_{k}+M^{2}\phi_{k})}{]}^{T}. \end{array}$$

For the MUSIC-based methods, the EVD is applied, and all the 2M+1 columns of C are taken into computation to get the two desired subspaces, which would of course reduce the computational efficiency. Instead, in this paper, we use only *K* columns, and the EVD can be avoided.

From Equation (17), we can see that each column of C is a linear combination of the columns of $A_{1}\left(\theta \right)$. The combination coefficients are the products of $C_{4s}A_{2}^{H}\left(\theta ,r\right)$. The 2M+1 columns of C and those of $A_{1}\left(\theta \right)$ span the same column subspace. Notice that the ranks of C and $A_{1}\left(\theta \right)$ are both *K*:

(21) $$rank\left(C\right)=rank(A_{1}\left(\theta \right))=K.$$

In order to improve the computational efficiency, let us take the first *K* columns of C to construct the following matrix:

(22) $$U_{s1}=\left[c_{1},c_{2},\ldots ,c_{K}\right].$$

In fact, any *K* different columns taken from C can be used to construct $U_{s1}$. The rank of $U_{s1}$ is *K* [35]. This is equivalent to the fact that the *K* columns of $U_{s1}$ and those of $A_{1}\left(\theta \right)$ span the same column subspace.

Define:

(23) $$U_{n1}=I-U_{s1}{\left(U_{s1}^{H}U_{s1}\right)}^{-1}U_{s1}^{H},$$

where I is an identity matrix with dimension (2M+1)×(2M+1). It is obvious that:

(24) $$\begin{array}{c} U_{s1}^{H}U_{n1}=0_{K\times (2M+1)}, \end{array}$$

where $0_{p\times q}$ is a p×q zero matrix. $U_{s1}$ is orthogonal to $U_{n1}$. As described previously, $U_{s1}$ and $A_{1}\left(\theta \right)$ span the same column subspace, which means that $A_{1}\left(\theta \right)$ is also orthogonal to $U_{n1}$. Then, we have:

(25) $$A_{1}^{H}\left(\theta \right)U_{n1}=0_{K\times (2M+1)}.$$

Consequently, the DOA estimation can be achieved with the following estimator:

(26) $${\hat{\theta }}_{k}=\arg\max_{\theta }\frac{1}{a_{1}^{H}\left(\theta \right)U_{n1}U_{n1}^{H}a_{1}\left(\theta \right)},\;\;k=1,\ldots ,K.$$

There would be *K* peaks corresponding to the *K* DOA estimates of the sources.

### 3.2. Range Estimation

Considering again the cumulant matrix in Equation (17): $C=A_{1}\left(\theta \right)C_{4s}A_{2}^{H}\left(\theta ,r\right)$; each row of C can be obtained through a linear combination of the rows of $A_{2}^{H}\left(\theta ,r\right)$.

Denote the first *K* rows of C by $r_{1}^{H},r_{2}^{H},\ldots ,r_{K}^{H}$. We can construct the following matrix:

(27) $$U_{s2}=\left[r_{1},r_{2},\ldots ,r_{K}\right].$$

The *K* columns of $U_{s2}$ and those of $A_{2}\left(\theta ,r\right)$ span the same column subspace.

Define:

(28) $$U_{n2}=I-U_{s2}{\left(U_{s2}^{H}U_{s2}\right)}^{-1}U_{s2}^{H}.$$

As before, we know that $A_{2}\left(\theta ,r\right)$ is orthogonal to $U_{n2}$:

(29) $$\begin{array}{c} A_{2}^{H}\left(\theta ,r\right)U_{n2}=0_{K\times (2M+1)}. \end{array}$$

Consequently, the kth range can be estimated by:

(30) $${\hat{r}}_{k}=\arg\max_{r}\frac{1}{a_{2}^{H}\left({\hat{\theta }}_{k},r\right)U_{n2}U_{n2}^{H}a_{2}\left({\hat{\theta }}_{k},r\right)},$$

where ${\hat{\theta }}_{k}$ is the kth estimated DOA in the previous operation. The obtained range estimate is then automatically paired with the substituted DOA estimate, and there is no need for the extra pairing algorithm.

The method in [34], SHO, needs to apply Gram–Schmidt orthogonalization to the first *K* eigenvectors to get the desired orthogonal matrix. However, the orthogonalization requires these *K* eigenvectors to be full column rank. This condition may not be satisfied in some special cases, which would limit the application of SHO.

In the proposed method, HOP, there is no constraint for the construction of $U_{n1}$ or $U_{n2}$. The limitation of SHO is avoided, and no EVD is required, reducing the computational complexity further.

The proposed method can be summarized as follows:

Step 1: Construct the cumulant matrix C.
Step 2: Obtain $U_{n1}$ orthogonal to $A_{1}\left(\theta \right)$.
Step 3: Estimate the DOAs ${\hat{\theta }}_{k}$ (k=1,2…,K).
Step 4: Calculate $U_{n2}$ orthogonal to $A_{2}\left(\theta ,r\right)$.
Step 5: Estimate the $k^{\mathrm{th}}$ range with the $k^{\mathrm{th}}$ estimated DOA.
Step 6: Repeat Step 5 until all the *K* range estimates are obtained.

### 3.3. Complexity Analysis

In this section, the computational complexity of the proposed method will be analyzed. In [34], the computational complexity of SHO has already been proven to be lower than that of MOS. In conclusion, the main complexities of SHO and HOP include the construction of the desired high-order cumulant matrix, the construction of the subspaces, and the spectrum searches, which are shown in Table 1.

Both methods need to build a (2M+1)×(2M+1) cumulant matrix, whose computational complexity is about $O(M^{2}T)$. K+1 spectrum searches are necessary to estimate the DOA and range, respectively. The computational complexity of one spectrum search is about $O(M^{2}N)$, where *N* is the number of grids for the search.

As for the different parts of the two methods, SHO needs to apply EVD to a (2M+1)×(2M+1) matrix, and the Gram–Schmidt orthogonalization is required. The computational complexity of these two steps is about $O(M^{3})$.

The proposed method, HOP, only needs to calculate two propagators in order to carry out the spectrum searches. The complexity of the propagator calculation is about $O(M^{2}K)$ [29].

In the case where the number of sources *K* is not much smaller than *M*, the complexities of HOP and SHO are similar. However, in most situations where there are only a few sources, HOP achieves a lower computational complexity than SHO.

## 4. Simulation Results and Analysis

In this section, we carry out several simulations to show the performance of the proposed method, compared with that of SHO [34], LOFNS [19], and the modified ESPRIT-like method [26].

### 4.1. Computational Efficiency

Firstly, the computational efficiency was assessed. We used a laptop as the platform, whose CPU was i5 (2.6 GHz) and RAM 6 GB. The simulation was carried out in the situation where there were two near-field sources. The array received 100 snapshots, and the number of grids for the spectrum search was 1080. In order to show the influence of the number of sensors, we ran the simulation with four different array configurations made of 5, 7, 9, and 11 sensors respectively. The efficiency of different methods was also shown in MATLAB flops versus the number of sensors, which can directly show the computational complexity.

**Figure 2:**
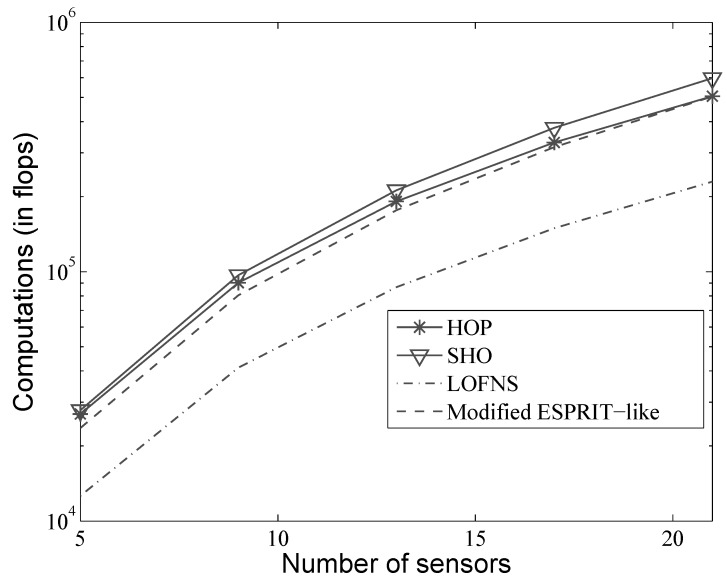
Flops versus number of sensors.

It should be noted that the spectrum search is necessary for SHO and HOP, while the modified ESPRIT-like and LOFNS are search-free methods. For SHO and HOP, we can see from Table 2 that when the number of sources is close to that of the sensors in the array, the subspace calculation is not a main factor of the computational efficiency. Other parts, such as the matrix construction, would play more important roles. In this case, the two methods perform almost the same, and the advantage of the subspace calculation of HOP is not sufficiently obvious. However, as the number of sensors increases, the way to calculate the subspace shows a difference in the computational efficiency. For the array made of 11 sensors, the average processing time of HOP is only about 70% of that of SHO. By using only K+1 columns and avoiding the EVD, propagator construction can significantly improve the efficiency, especially when the number of sensors is much bigger than that of the sources, which is also revealed in Figure 2. For the search-free methods, LOFNS uses second-order statistics and applies no EVD or SVD, achieving a better computational efficiency than the modified ESPRIT-like method.

### 4.2. Resolution Probability

Secondly, we examined the resolution probability when the signal-to-noise radio (SNR) varies. The SNR is defined as follows:

(31) $$SNR=10\log_{10}\frac{\sum_{k=1}^{K}P_{s_{k}}}{\sigma^{2}},$$

where $\sigma^{2}$ is the noise variance and $P_{s_{k}}$ the power of the kth signal.

The resolution probability is defined as the ratio between the successful estimation number and the number of total experiments. For the situation where there are two sources, the ith estimation is considered to be successful if the following condition is satisfied:

(32) $$|{\hat{\theta }}_{i}-\theta_{true}|<\frac{\Delta \theta }{2},$$

where ${\hat{\theta }}_{i}$ is the estimate of the ith trial, $\theta_{true}$ is the true value, and $\Delta \theta =|\theta_{1}-\theta_{2}|$.

Suppose there are two sources located at $\left[5^{\circ },2.8\lambda \right]$ and $\left[10^{\circ },2.8\lambda \right]$. The array was composed of seven sensors, and 500 snapshots were received by the array. Two thousand independent Monte Carlo simulations were run to get the resolution probability.

**Figure 3:**
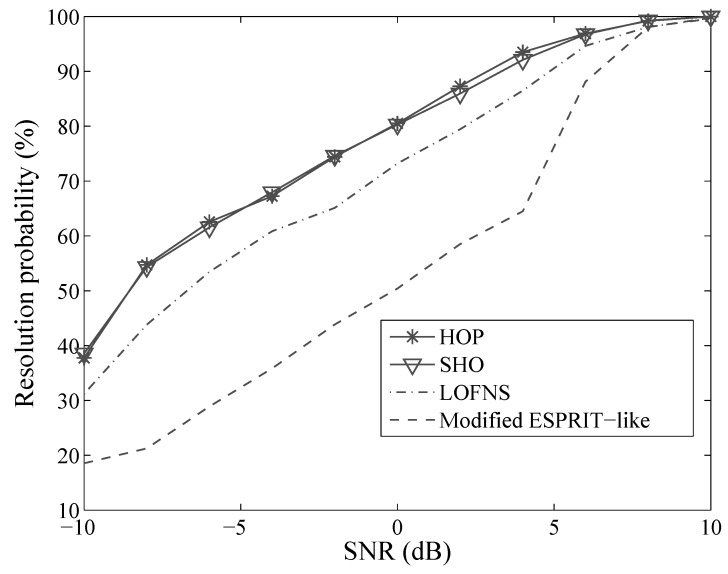
Resolution probability versus SNR.

Figure 3 shows the resolution probability of the four methods. These methods can resolve sources better as the SNR grows. SHO and HOP outperformed LOFNS and the modified ESPRIT-like method. For LOFNS, the size of the matrix for DOA estimation was only (M+1)×(M+1). The performance suffered from aperture loss, compared with the number of the real sensors in the ULA, which was 2M+1. SHO and HOP provided almost the same performance. Even when the SNR was 0 dB, the resolution probabilities were about 80%, and both of them reached 100% when the SNR was around 9 dB.

### 4.3. RMSE

This part will study the relationship between the root mean squared error (RMSE) of estimation and the SNR. The RMSE is defined as follows:

(33) $$RMSE=\sqrt{\frac{\sum_{p=1}^{P}|{\hat{\alpha }}_{p}-\alpha_{true}{|}^{2}}{P}},$$

where ${\hat{\alpha }}_{p}$ is the estimation result of the pth trial, $\alpha_{true}$ is the true value, and *P* is the number of independent Monte Carlo trials.

**Figure 4:**
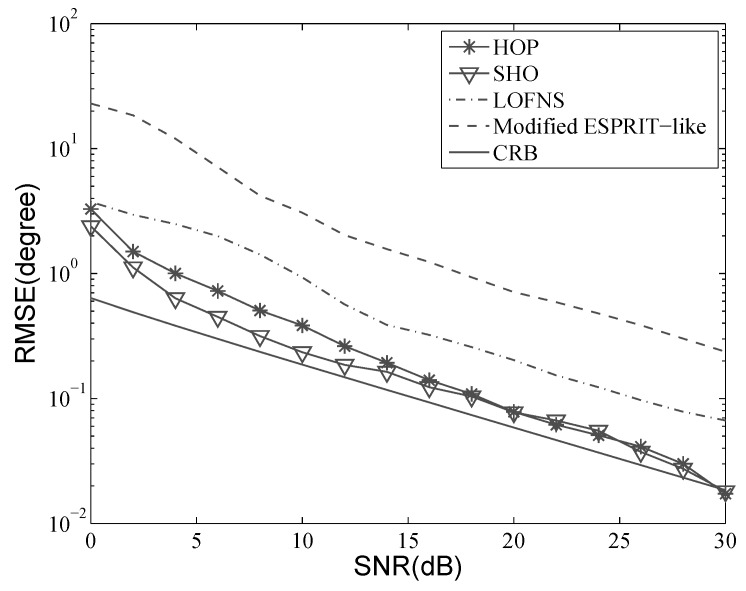
RMSE versus SNR: DOA of the first source.

**Figure 5:**
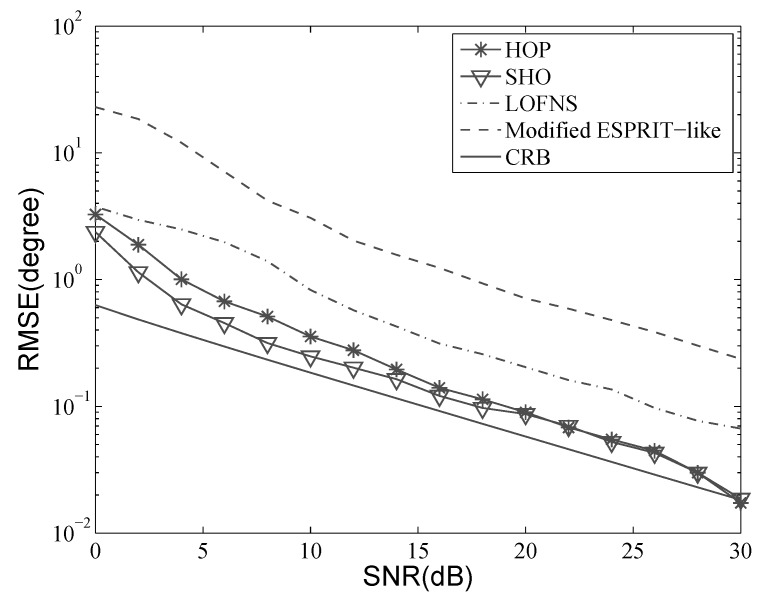
RMSE versus SNR: DOA of the second source.

**Figure 6:**
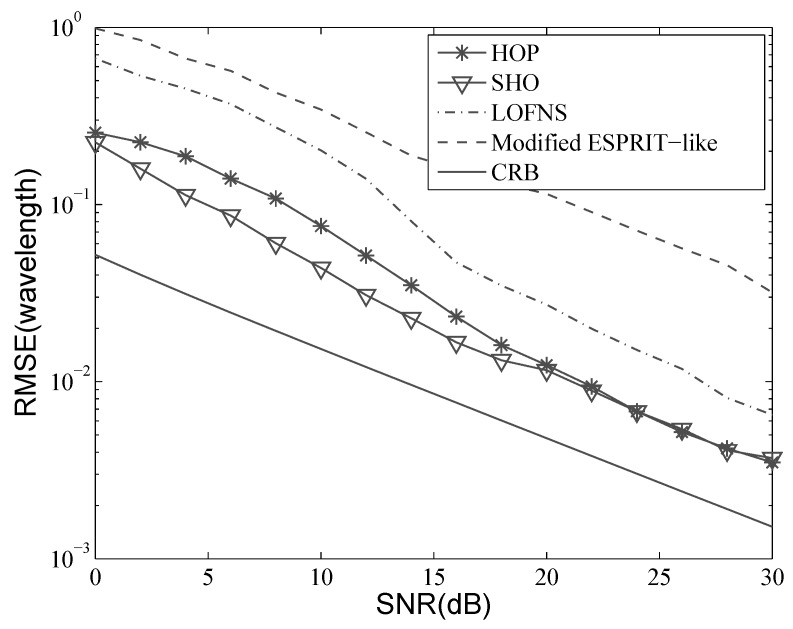
RMSE versus SNR: range of the first source.

**Figure 7:**
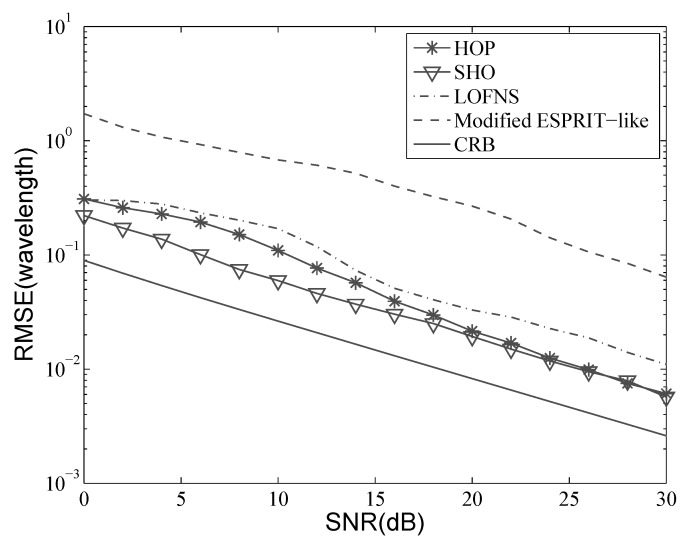
RMSE versus SNR: range of the second source.

Consider the situation where two sources are located at $\left[-10^{\circ },1.5\lambda \right]$ and $\left[5^{\circ },2.0\lambda \right]$. A ULA with five sensors was used, and the inter-element distance *d* was $\frac{\lambda }{4}$. Five hundred snapshots were used in the simulation. Let the SNR vary from 0 dB–30 dB; the results with 500 independent Monte Carlo trials are shown in Figure 4, Figure 5, Figure 6 and Figure 7. Besides, the performance of the proposed method was also compared with the Cramer–Rao bound (CRB).

The CRB is a theoretical lower bound revealing the best performance that any unbiased estimator could achieve [36]. In order to get the CRB, the Fisher information matrix (FIM) needs to be calculated first. Grosicki et al. derived the analytical expression of the CRB for near-field source localization [18], which is very practical to evaluate the performance of different unbiased estimators.

Figure 4 and Figure 5 show that the RMSE of DOA decreased when SNR increased. The estimation accuracies of HOP and SHO were better than those of LOFNS and the modified ESPRIT-like method. The aperture loss of LOFNS discussed in Section 4.2 affected not only the resolution probability, but also the estimation accuracy. Thanks to the resistance to Gaussian noise of the high-order cumulant, the results of HOP and SHO were very close to CRB and were getting closer as the SNR was getting bigger. Comparing Figure 4 with Figure 5, we can also see that the accuracies of DOA estimation were almost the same for different sources.

As we can see, HOP did not perform as well as SHO when the SNR was low. SHO applies the EVD with all the 2M+1 columns, while HOP uses only *K* columns to calculate propagators, which can greatly improve the computational efficiency. However, this improvement is at the cost of reduced estimation precision [30]. When the SNR became bigger than 15 dB, the two methods performed similarly, and the negative effect of the proposed method could be negligible.

Figure 6 and Figure 7 illustrate the results of the range estimation. From the definition of $\phi_{k}$, we know that the range estimation relies on that of DOA, if we estimate the two parameters in a decoupled way. The DOA estimation needs to be accomplished before the range estimation, and the accuracy of DOA estimate will directly impact that of the range estimate. Therefore, the range estimation accuracy of LOFNS is not as good as that of SHO and HOP, even though the range estimation in LOFNS does not suffer from aperture loss.

For SHO and HOP, we can see that when the SNR was low, SHO outperformed HOP, which agrees well with the previous analysis. When the SNR was bigger than 20 dB, the drawback of HOP had little impact on the estimation results.

We can also see from Figure 6 and Figure 7 that the accuracy of the range estimation varied for different sources. The accuracy depends on the distance between the source and the array. The accuracy will get higher as the source gets closer to the array, which has been pointed out in the theoretical analysis in [37].

## 5. Conclusions

In this paper, we have proposed a new efficient high-order propagator-based method (HOP) to localize near-field sources. After constructing a specific cumulant matrix, we show that the DOAs and ranges of near-field sources can be estimated in a decoupled way with only one single matrix and without the EVD. Simulation results show the efficiency of the proposed method, which achieves a lower computational complexity than SHO, while performing almost as well as other high-order methods. The lower computational complexity would make an important contribution to portable detecting devices, especially for long-time field applications, where the electronic energy cannot be fully guaranteed. Besides, SHO requires the constructed matrix to be diagonalizable. This constraint no longer holds for HOP, leading to its wider applications.

## Figures and Tables

**Table 1:** Main complexities of different methods. SHO, simplified high-order MUSIC method; HOP, high-order propagator-based method.

Item	SHO	HOP
Matrix construction	1	1
EVD	1	0
Gram-Schmidt orthogonalization	1	0
Number of calculated columns	2M+1	*K*
Propagator construction	0	2
Spectrum search	K+1	K+1

**Table 2:** Average processing time (seconds) for different methods.

Number of Sensors	5	7	9	11
SHO	0.10928	0.13108	0.16641	0.21607
HOP	0.11337	0.12281	0.14387	0.15294
Modified ESPRIT-like	0.06708	0.08979	0.10404	0.13442
LOFNS	0.03279	0.04108	0.06376	0.08135

## Abbreviations

The following abbreviations are used in this manuscript:

DOA	Direction of arrival
2D	Two-dimensional
MUSIC	Multiple signal classification
ESPRIT	Estimation of signal parameters via rotation-invariant technique
EVD	Eigenvalue decomposition
SVD	Singular-value decomposition
ULA	Uniform linear array
CPU	Central processing unit
RAM	Random access memory
SNR	Signal-to-Noise Ratio
RMSE	Root mean squared error
CRB	Cramer–Rao bound
FIM	Fisher information matrix
